# Hyperglycaemia increases mortality risk in non‐diabetic patients with COVID‐19 even more than in diabetic patients

**DOI:** 10.1002/edm2.291

**Published:** 2021-07-18

**Authors:** Jennifer Morse, Wendy Gay, Kimberly M. Korwek, Laura E. McLean, Russell E. Poland, Jeffrey Guy, Kenneth Sands, Jonathan B. Perlin

**Affiliations:** ^1^ Clinical Operations Group HCA Healthcare Nashville TN USA

**Keywords:** COVID‐19, glucose variability, hyperglycaemia, inpatient glycaemic management

## Abstract

**Aim:**

Diabetes has been identified as a risk factor for poor outcomes in patients with COVID‐19. We examined the association of hyperglycaemia, both in the presence and absence of pre‐existing diabetes, with severity and outcomes in COVID‐19 patients.

**Methods:**

Data from 74,148 COVID‐19‐positive inpatients with at least one recorded glucose measurement during their inpatient episode were analysed for presence of pre‐existing diabetes diagnosis and any glucose values in the hyperglycaemic range (>180 mg/dl).

**Results:**

Among patients with and without a pre‐existing diabetes diagnosis on admission, mortality was substantially higher in the presence of high glucose measurements versus all measurements in the normal range (70–180 mg/dl) in both groups (non‐diabetics: 21.7% vs. 3.3%; diabetics 14.4% vs. 4.3%). When adjusting for patient age, BMI, severity on admission and oxygen saturation on admission, this increased risk of mortality persisted and varied by diabetes diagnosis. Among patients with a pre‐existing diabetes diagnosis, any hyperglycaemic value during the episode was associated with a substantial increase in the odds of mortality (OR: 1.77, 95% CI: 1.52–2.07); among patients without a pre‐existing diabetes diagnosis, this risk nearly doubled (OR: 3.07, 95% CI: 2.79–3.37).

**Conclusion:**

This retrospective analysis identified hyperglycaemia in COVID‐19 patients as an independent risk factor for mortality after adjusting for the presence of diabetes and other known risk factors. This indicates that the extent of glucose control could serve as a mechanism for modifying the risk of COVID‐19 morality in the inpatient environment.

## Graphical abstract

This retrospective analysis identified hyperglycaemia in COVID‐19 patients as a risk factor for mortality after adjusting for the presence of diabetes and other known risk factors. Among nearly 75,000 COVID‐19 patients with and without a pre‐existing diabetes diagnosis on admission, mortality was substantially higher in the presence of high glucose measurements versus all measurements in the normal range (70–180 mg/dl) in both groups (non‐diabetics: 21.7% vs. 3.3%; diabetics 14.4% vs. 4.3%). This indicates that effective glucose control could serve as a mechanism for modifying the risk of COVID‐19 morality.

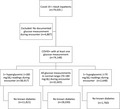

## 1 INTRODUCTION

COVID‐19 disease, caused by the novel severe acute respiratory syndrome‐associated coronavirus‐2 (SARS‐CoV‐2), surpassed 22 million cases in the United States and over 370,000 deaths in January 2021.^1^ While any patient can be at risk for severe complications and mortality due to COVID‐19, considerable attention has been paid to identification of potential risk factors. Older age (>65 years), male gender and comorbidities such as hypertension and diabetes have been consistently identified as increasing the risk for more severe disease course or mortality.^2^

While the presence of diabetes is more commonly associated with severe COVID‐19, the mechanism by which this occurs remains to be elucidated.^3^ In general, glycaemic control in the inpatient environment has been linked to outcomes in critically ill patients, regardless of diabetes status.^4^, ^5^, ^6^, ^7^ Indeed, increased peak glucose values and increased time in a hyperglycaemic range appear to be associated with increased mortality among patients without pre‐existing diabetes but not those with the condition.^8^ While exact glucose targets and the importance of tight glucose control have varied based on study results, the current guidelines for inpatient glycaemic management are consistent with long‐standing evidence‐based best practices. Guidelines recommend that hyperglycaemia (readings >140) first be treated conservatively with the addition of the use of insulin to control of blood glucose when levels reach 180 mg/dl in the inpatient environment for all patients.^9^, ^10^

In critically ill COVID‐19 patients specifically, both a decreased time in a normoglycaemic range and increased insulin requirements in comparison to non‐COVID‐19 patients have been reported; this has been associated with an increase in ventilator use and mortality among these patients.^11^ The acute hyperglycaemia and insulin resistance that is common in critically ill patients in addition to the increasing use of steroid treatment for COVID‐19 complicate the control of blood sugar in these patients.^12^, ^13^

Control of blood glucose among COVID‐19 patients with pre‐existing diabetes has been correlated with better outcomes.^14^, ^15^ Furthermore, more recent evidence has begun to suggest that hyperglycaemia, independent of diabetes, is an independent risk factor for more severe COVID‐19 disease course and outcomes.^16^, ^17^ Thus, hyperglycaemia may represent a risk factor for COVID‐19 outcomes that could be modifiable with improved glucose control.

In this retrospective analysis of nearly 75,000 COVID‐19‐positive inpatients in the United States, we examined the relationship between hyperglycaemia over the course of the entire episode and discharge disposition. An understanding of how hyperglycaemia affects outcomes in COVID‐19 patients, both with and without a previous diabetes diagnosis, will provide insight into potential care improvement for these patients.

## 2 MATERIALS AND METHODS

Data used in this study were collected in the electronic health records during the care of patients admitted to facilities affiliated with a large healthcare system in the United States from 4 December 2019 to 7 December 2020. Only patients with a documented discharge disposition were included. Patients admitted directly to hospice were excluded. Patients were deemed COVID‐19 positive (COVID‐19+) if there was documentation of at least one positive detection of SARS‐CoV‐2 by PCR, either immediately prior to or during the hospital stay.

COVID‐19‐positive inpatients were included in the analysis if they had at least one glucose measurement recorded during their stay. Measurements were included from both point of care and laboratory results. Glucose values were categorized by the following criteria: hypoglycaemia, <70 mg/dl; normal, 70–180 mg/dl; and hyperglycaemia, >180 mg/dL.^18^ Comorbidities, including diabetes diagnosis, were determined using ICD‐10 codes from present on admission diagnoses.

### 2.1 Statistical methods

The primary outcome of interest was mortality. Logistic regression was used to estimate the association between a patient's glycaemic metric and odds of mortality for the previously defined COVID‐19 patient population. The adjusted regression controlled for diabetes, age, body mass index (BMI), severity and oxygen saturation at admission. Covariates in the model were defined as follows: diabetes: binary variable based on final diagnosis of diabetes from verified list of ICD‐10 codes; age: numeric years at discharge, treated as cubic spline; BMI: numeric average throughout stay, treated as cubic spline; severity: categorical variable based on level of service (either within 8 h of admission or overall), levels defined as medical/surgical (no ICU or mechanical ventilation), ICU, or mechanical ventilation; oxygen saturation at admission: first reading upon admission, treated as cubic spline. A sensitivity analysis was performed using multiple imputation to account for missing data (10%–20%); model estimates and confidence intervals were similar and results unchanged.

## 3 RESULTS

**FIGURE 1:**
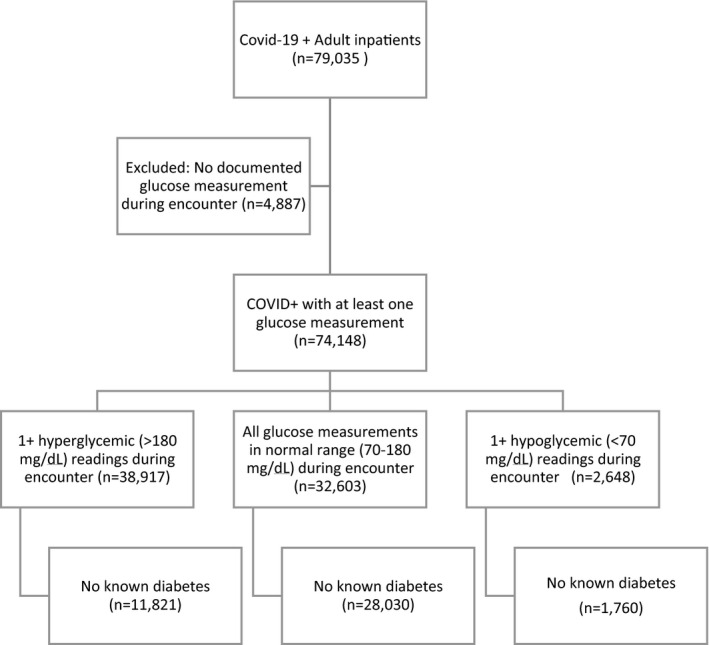
Analysis data set diagram

As of 7 December 2020, there were 79,035 adult COVID‐19+ inpatients with documented discharge disposition. Of those, 74,148 (94%) patients had at least one glucose measurement recorded during their episode and were thus included in the final data set (Figure 1). Of these COVID‐19+ inpatients with at least one glucose measurement, 38,917 (53%) had one or more glucose readings above 180 mg/dl (defined as hyperglycaemia in this study). This group included 11,821 (30%) with no pre‐existing diagnosis of diabetes; 27,096 (70%) of patients with at least one hyperglycaemic reading during their episode had a diagnosis of diabetes at admission.

Mortality among COVID‐19+ inpatients with one or more glucose measurements in the hyperglycaemic range (>180 mg/dl) was 16.6%; in comparison, mortality among COVID‐19+ inpatients with all glucose measurements in the normal range during their entire stay (70–180 mg/dl) was 3.3% (Table 1). Mortality increased with additional hyperglycaemic measurements, but the majority of patients with at least one measurement in the hyperglycaemic range had a subsequent hyperglycaemic reading. Patients with only one hyperglycaemic measurement over the course of the encounter (*n* = 602) had a mortality of 10.6%. Those with only two hyperglycaemic measures (n = 398) had a mortality of 13.6%; only three (*n* = 310), 14.3% mortality and four or more (*n* = 5162), 18.4%.

**TABLE 1:** Patient characteristics by presence of hyperglycaemia during stay

	All glucose measurements in normal range (70–180 mg/dl) (*n* = 32,603)	1+ high glucose measurements (>180 mg/dl) (*n* = 38,917)
Age in years, mean (SD)	60.8 (19.3)	64.7 (15.2)
Sex: Male (%)	51.0	54.6
Race: White (%)	49.2	45.3
BMI, mean (SD)	30.3 (8.9)	31.6 (8.7)
Pre‐existing diabetes diagnosis (%)	14.0%	69.6%
Number of glucose tests, mean (SD)	6.5 (10.0)	47.3 (72.1)
LOS in days, median [IQR]	**4 [2–7]**	**8 [4–14]**
Mortality (%)	3.3	16.6
Mortality, diabetic	4.3	14.4
Mortality, non‐diabetic	3.3	21.7
Severity (Level of service) at admission		
Medical/Surgical	85.6	66.7
ICU	10.9	12.9
Mechanical Ventilation	3.5	20.5
Highest severity (level of service) during episode		
Medical/Surgical	80.4	57.6
ICU	16.1	21.9
Mechanical Ventilation	3.5	20.5

As expected, there was a high proportion of patients with pre‐existing diabetes (69.6%) in the group with documented hyperglycaemia during their episode. Mortality was higher in patients with diabetes than in non‐diabetics whether hyperglycaemia was present or not. However, among patients with and without a pre‐existing diabetes diagnosis, mortality was substantially higher in the presence of high glucose measurements versus all measurements in the normal range (non‐diabetics: 21.7% vs. 3.3%; diabetics 14.4% vs.d 4.3%); see Table 1.

Glucose variability during the encounter, defined as one or more glucose measurements less than 70 mg/dl and one or more measurements greater than 180 mg/dl, was observed in 12.0% of patients in the data set (*n* = 8889). Mortality in this group was 25.7% overall; mortality was higher among those without a pre‐existing diabetes diagnosis (diabetics: 22.8%; non‐diabetics: 37.2%).

Logistic regression was used to estimate the odds of mortality with one or more hyperglycaemic values during the episode for COVID‐19 patients both with and without a history of diabetes. In the unadjusted model, the odds of mortality were increased with the presence of one or more glucose values >180 mg/dl (OR: 4.85, 95% CI: 4.57–5.15). After adjusting for patient age, BMI, severity on admission and oxygen saturation on admission, this increased risk of mortality persisted and varied by diabetes diagnosis. Among patients with a pre‐existing diabetes diagnosis, any hyperglycaemic value during the episode was associated with a substantial increase in the odds of mortality compared to diabetic patients without hyperglycaemia (OR: 1.77, 95% CI: 1.52–2.07); among patients without a pre‐existing diabetes diagnosis, this risk nearly doubled (OR: 3.07, 95% CI: 2.79–3.37); see Table 2.

**TABLE 2:** Odds of mortality by presence of diabetes and hyperglycaemia

	Pre‐existing diabetes			No known diabetes		
	N	Per cent mortality (Raw)	Adjusted Odds Ratio* [95% CI]	N	Per cent mortality (Raw)	Adjusted Odds Ratio [95% CI]
Reference (no values >180 mg/dl)^†^	5,448	5.2%	–	29,783	3.7%	–
1+ high value (>180 mg/dl)	27,096	14.4%	1.77 [1.52– 2.07]	11,821	21.7%	3.07 [2.79–3.37]

* Note: Model is adjusted for age, BMI, severity, SpO_2_ on admission and diabetes.

† Reference may include patients with values <70 mg/dL during stay.

## 4 DISCUSSION

In this analysis of nearly 75,000 COVID‐19 patients, the presence of hyperglycaemia (blood glucose >180 mg/dl) at any point during the inpatient episode was associated with a substantial increase in the odds of mortality, regardless of pre‐existing diabetes diagnosis. This increased risk persisted after adjusting for known influencers of COVID‐19 outcomes, such as age, BMI, severity and oxygen saturation at admission. While diabetes has been highlighted as a covariate that increases the risk of poor outcomes with COVID‐19, we observed that hyperglycaemia appears to be a potentially modifiable risk factor for mortality.

Altered glucose metabolism, including hyperglycaemia, is common among patients with any critical illness and is associated with increased risk of mortality.^8^, ^19^ Given this known relationship between hyperglycaemia and critical illness, our similar findings in COVID‐19 patients are not surprising. What is interesting is the substantial increased effect of hyperglycaemia on the risk of mortality among patients without a pre‐existing diabetes diagnosis. While guidelines recommend awareness and maintenance of glycaemic control for all inpatients, especially the critically ill, anecdotally, much of the attention regarding maintenance of normoglycaemia in the inpatient environment is focussed on patients with diabetes due to concerns related to overtreatment and resulting hypoglycaemia.^6^, ^9^ However, our findings add to increasing evidence that suggests that the bodies of patients with diabetes are more accustomed to hyperglycaemia and thus may be better able to withstand the effects of acute hyperglycaemia during critical illnesses such as COVID‐19.^12^, ^20^

Alternatively, patients with diabetes may be receiving more aggressive management of hyperglycaemia during their episode, as these patients and their providers are more attune to their blood glucose levels and importance of managing this chronic condition in the acute care environment. As expected, our data confirm the association among diabetes diagnosis, in‐hospital hyperglycaemia and mortality. Additional analysis is necessary to determine the effect of insulin treatment on outcomes in COVID‐19 patients and the interaction with previous diabetes diagnoses and hyperglycaemia. However, it is notable that even with adjustments for diabetes diagnosis, age and other comorbidities, the presence of any instance of hyperglycaemia is associated with an increased risk for mortality in COVID‐19+ inpatients.

Glucose management in COVID‐19 patients is dually complicated by the disease itself and by the current standards of care. Due to the ability of coronaviruses to bind to the angiotensin‐converting enzyme 2 (ACE2) receptors expressed in metabolic organs, it is plausible that early hyperglycaemia in COVID‐19+ patients could be related to the disease course itself.^21^ Indeed, there have been several reports indicating that COVID‐19 may be associated with alterations to glucose metabolism.^22^, ^23^ The increased use of steroids as standard treatment for COVID‐19 patients also adds further complexity to glucose control in these patients; while the majority of patients in our data set received some type of steroid over the course of their episode, additional analysis is in progress to determine whether there is additional interaction between this treatment, hyperglycaemia and outcomes.^24^, ^25^

The retrospective nature of this data analysis limits our conclusions to detection of correlation, not causality. The data set used herein identified diabetes as coded in the medical record. Thus, it is possible that a portion of the population identified as non‐diabetic in this study could consist of patients in which diabetes had not yet been identified or diagnosed. As haemoglobin A1C levels are not commonly obtained in the acute care environment, especially during COVID‐19 surge conditions, it will be difficult to separate out those patients with pre‐existing, non‐diagnosed diabetes from those that develop alterations in glucose metabolism during the disease course in this particular data set. Additional analysis is underway to determine how COVID‐19 treatments and the management of hyperglycaemia interact to influence patient outcomes.

Taken together, this analysis emphasizes the importance of glucose management in patients with COVID‐19, especially among those without a pre‐existing diabetes diagnosis. Anecdotal reports from diabetes care leaders at affiliated facilities and preliminary analysis of insulin administration data suggest that a substantial proportion of the glucose management in COVID‐19 patients is being done via subcutaneous insulin and that there are fewer orders for point of care monitoring of glucose values. This may be due to concerns about employee exposure and the availability of personal protective gear. Regardless of the mechanism, this type of glucose management poses additional challenges, as guidelines recommend the use of intravenous insulin to control blood glucose levels in critically ill patients or basal‐bolus insulin therapy in non‐ICU environments, as blood glucose is more easily maintained by these mechanisms.^9^

In total, this retrospective analysis identified hyperglycaemia in COVID‐19 patients as a risk factor for mortality after adjusting for the presence of diabetes and other known risk factors. This suggests that effective glucose control could serve as a means for modifying the risk of COVID‐19 morality and that providers should consider ways to incorporate this into the care of COVID‐19 patients to potentially reduce hyperglycaemia and improve outcomes.

## CONFLICT OF INTEREST

All authors declare no conflicts of interest.

## AUTHOR CONTRIBUTIONS

J.M researched data, W.G. contributed to the discussion and reviewed/edited the manuscript, K.K. contributed to the discussion and wrote the manuscript, L.M. researched data, R.P contributed to the discussion and reviewed/edited the manuscript, J.G contributed to the discussion and reviewed/edited the manuscript, K.S. contributed to the discussion and reviewed/edited the manuscript, J.P contributed to the discussion and reviewed/edited the manuscript.

## Data Availability

The data that support the findings of this study are available from the corresponding author upon reasonable request.

## References

[1] Centers for Disease Control and Prevention . Coronavirus Disease 2019 (COVID‐19) Cases in U.S. 2020; https://www.cdc.gov/coronavirus/2019‐ncov/cases‐updates/cases‐in‐us.html. Accessed April 22, 2020.

[2] Wolff D , Nee S , Hickey NS , Marschollek M . Risk factors for Covid‐19 severity and fatality: a structured literature review. Infection. 2020;49(1):15‐28. 32860214 10.1007/s15010-020-01509-1 PMC7453858

[3] Apicella M , Campopiano MC , Mantuano M , Mazoni L , Coppelli A , Del Prato S . COVID‐19 in people with diabetes: understanding the reasons for worse outcomes. Lancet Diabetes Endocrinol. 2020;8(9):782‐792. 32687793 10.1016/S2213-8587(20)30238-2 PMC7367664

[4] Umpierrez GE , Isaacs SD , Bazargan N , You X , Thaler LM , Kitabchi AE . Hyperglycemia: an independent marker of in‐hospital mortality in patients with undiagnosed diabetes. J Clin Endocrinol Metab. 2002;87(3):978‐982. 11889147 10.1210/jcem.87.3.8341

[5] Dungan KM , Braithwaite SS , Preiser JC . Stress hyperglycaemia. Lancet. 2009;373(9677):1798‐1807. 19465235 10.1016/S0140-6736(09)60553-5 PMC3144755

[6] The NICE‐SUGAR Study Investigators . Intensive versus conventional glucose control in critically Ill patients. N Engl J Med. 2009;360(13):1283‐1297. 19318384 10.1056/NEJMoa0810625

[7] Bellaver P , Schaeffer AF , Dullius DP , Viana MV , Leitão CB , Rech TH . Association of multiple glycemic parameters at intensive care unit admission with mortality and clinical outcomes in critically ill patients. Sci Rep. 2019;9(1):18498. 31811218 10.1038/s41598-019-55080-3 PMC6897941

[8] Plummer MP , Bellomo R , Cousins CE , et al. Dysglycaemia in the critically ill and the interaction of chronic and acute glycaemia with mortality. Intensive Care Med. 2014;40(7):973‐980. 24760120 10.1007/s00134-014-3287-7

[9] Moghissi ES , Korytkowski MT , DiNardo M , et al. American Association of Clinical Endocrinologists and American Diabetes Association consensus statement on inpatient glycemic control. Diabetes Care. 2009;32(6):1119‐1131. 19429873 10.2337/dc09-9029 PMC2681039

[10] American Diabetes Association . Diabetes care in the hospital: standards of medical care in diabetes—2020. Diabetes Care. 2020;43(Supplement 1):S193‐S202. 31862758 10.2337/dc20-S015

[11] Kapoor R , Timsina LR , Gupta N , et al. Maintaining blood glucose levels in range (70–150 mg/dL) is difficult in COVID‐19 compared to Non‐COVID‐19 ICU patients‐a retrospective analysis. J Clin Med. 2020;9(11):70‐150. 10.3390/jcm9113635 PMC7697842 33198177

[12] Egi M , Bellomo R , Stachowski E , et al. Blood glucose concentration and outcome of critical illness: the impact of diabetes. Crit Care Med. 2008;36(8):2249‐2255. 18664780 10.1097/CCM.0b013e318181039a

[13] Gianchandani R , Esfandiari NH , Ang L , et al. Managing hyperglycemia in the COVID‐19 inflammatory storm. Diabetes. 2020;69(10):2048‐2053. 32778570 10.2337/dbi20-0022

[14] Zhu L , She Z‐G , Cheng X , et al. Association of blood glucose control and outcomes in patients with COVID‐19 and pre‐existing type 2 diabetes. Cell Metab. 2020;31(6):1068‐1077.e1063. 32369736 10.1016/j.cmet.2020.04.021 PMC7252168

[15] Hill MA , Mantzoros C , Sowers JR . Commentary: COVID‐19 in patients with diabetes. Metabolism. 2020;107:154217. 32220611 10.1016/j.metabol.2020.154217 PMC7102643

[16] Bode B , Garrett V , Messler J , et al. Glycemic characteristics and clinical outcomes of COVID‐19 patients hospitalized in the United States. J Diabetes Sci Technol. 2020;14(4):813‐821. 32389027 10.1177/1932296820924469 PMC7673150

[17] Coppelli A , Giannarelli R , Aragona M , et al. Hyperglycemia at hospital admission is associated with severity of the prognosis in patients hospitalized for COVID‐19: the Pisa COVID‐19 study. Diabetes Care. 2020;43(10):2345‐2348. 32788285 10.2337/dc20-1380

[18] American Diabetes Association . Diabetes care in the hospital. Diabetes Care. 2016;39(Supplement 1):S99‐S104. 26696689 10.2337/dc16-S016

[19] Cely CM , Arora P , Quartin AA , Kett DH , Schein RM . Relationship of baseline glucose homeostasis to hyperglycemia during medical critical illness. Chest. 2004;126(3):879‐887. 15364770 10.1378/chest.126.3.879

[20] Rady MY , Johnson DJ , Patel BM , Larson JS , Helmers RA . Influence of individual characteristics on outcome of glycemic control in intensive care unit patients with or without diabetes mellitus. Mayo Clin Proc. 2005;80(12):1558‐1567. 16342648 10.4065/80.12.1558

[21] Ni W , Yang X , Yang D , et al. Role of angiotensin‐converting enzyme 2 (ACE2) in COVID‐19. Crit Care. 2020;24(1):422. 32660650 10.1186/s13054-020-03120-0 PMC7356137

[22] Rubino F , Amiel SA , Zimmet P , et al. New‐onset diabetes in Covid‐19. N Engl J Med. 2020;383(8):789‐790. 32530585 10.1056/NEJMc2018688 PMC7304415

[23] Boddu SK , Aurangabadkar G , Kuchay MS . New onset diabetes, type 1 diabetes and COVID‐19. Diabetes Metab Syndr. 2020;14(6):2211‐2217. 33395782 10.1016/j.dsx.2020.11.012 PMC7669477

[24] The RECOVERY Collaborative Group . Dexamethasone in hospitalized patients with Covid‐19 — preliminary report. N Engl J Med. 2021;384(8):693‐704. 32678530 10.1056/NEJMoa2021436 PMC7383595

[25] Qi D , Pulinilkunnil T , An D , et al. Single‐dose dexamethasone induces whole‐body insulin resistance and alters both cardiac fatty acid and carbohydrate metabolism. Diabetes. 2004;53(7):1790‐1797. 15220203 10.2337/diabetes.53.7.1790

